# Personal

**Published:** 1908-06

**Authors:** 


### PERSONAL.

Dr. Sydney A. Dunham, of Buffalo, is in Kansas City attending the Presbyterian general assembly which convened May 21, and will hold until May 30. Dr. Dunham will then attend as a delegate the meeting of the American Medical Association, which will be held in Chicago from June 2 to June 5, 1908.

Dr. James A. MacLeod, of Buffalo, will remove his office and residence from 262 to 327 Delaware Avenue on June 1, 1908. His brother, Dr. Norman K. MacLeod, formerly of Toronto, will be associated with him in practice. Dr. Norman K. MacLeod will also give special attention to laboratory work in bacterial vaccines.

Dr. H. W. Cowper, of Buffalo, announces to the profession that he has established offices at 385 Franklin Street, Buffalo. His practice is confined to ophthalmology.

Dr. Carl G. Leo-Wolf, of Niagara Falls, N. Y., announces under date of May 15, 1908, that on or about May 20, he will go abroad for two or three months to study at the European clinics. During his absence Dr. Otto R. Eichel, late house-surgeon at the Buffalo General Hospital, will occupy his offices and attend to his practice. Dr. Eichel's residence will be in the Y. M. C. A. building. Office: Rooms 300, 301, 302, 44 Falls St. Telephones for office and residence, Bell 244, Home 254.

Dr. William Gaertner, of Buffalo, has removed his office and residence from 602 Ellicott street to 194 East Utica street.

Dr. Joseph Burke, of Buffalo, has removed his office and residence from 888 Main to 1092 Main street.

Dr. John B. Murphy, of Chicago, has resigned as Professor of Surgery and co-head of the Department in Rush Medical College and has accepted the professorship of surgery and head of the department in Northwestern University Medical School and position of attending surgeon at Mercy Hospital.

---

Dr. A. W. Meyer, of the University of Minnesota and formerly of Johns Hopkins has accepted the professorship of anatomy in Northwestern University Medical School.

---

Dr. A. N. Richards, of the College of Physicians and Surgeons of New York City, has been appointed professor of pharmacology in Northwestern University Medical School.

---

Dr. Alexander T. Bull, of Buffalo, returned recently from a vacation of six weeks spent at Cambridge Springs in celebration of an event that few physicians can count—the completion of his sixtieth year of continuous service as a physician. Dr. Bull graduated in a class of 33 at the New York University in 1848, and went at once into practice at Quarantine, New York. It was a cholera year and three doctors had died within 30 days at the post he took. He was then 21 years old. Within a few months Dr. Bull showed unmistakable evidences of tuberculosis, of which many members of his family had died. He was ordered to the Delaware Mountains, the Adirondacks of those days, and spent three years in the wilderness of Sullivan county. At that time there was not a drug store in the county. Fresh air and outdoor life restored his health and today he is hale and clear-headed at 81 years and has the carriage of a grenadier. Dr. Bull has long held a prominent rank among the older homeopathic physicians in the State. He has lived to see the "old school" and the "new" united in many ways and co-operating successfully in abating the thousand ills that attack humankind.

---

Dr. F. Park Lewis, of Buffalo, chairman of the committee on ophthalmia neonatorum of the American Medical Association, will present the report of his committee to the house of delegates at Chicago, Monday, June 1, 1908. The report is published in full in the Journal of the American Medical Association, May 23, 1908, and should be read by every physician. Its importance cannot be overestimated.

